# Association between pertussis vaccination coverage and other sociodemographic factors and pertussis incidence using surveillance data

**DOI:** 10.1016/j.epidem.2023.100689

**Published:** 2023-05-18

**Authors:** Madhura S. Rane, Jonathan Wakefield, Pejman Rohani, M. Elizabeth Halloran

**Affiliations:** a Department of Epidemiology, University of Washington, Seattle, WA 98195, USA; b Department of Biostatistics, University of Washington, Seattle, WA 98195, USA; c Department of Statistics, University of Washington, Seattle, WA 98195, USA; d Odum School of Ecology, University of Georgia, Athens, GA, 30602, USA; e Department of Infectious Diseases, University of Georgia, Athens, GA, 30602, USA; f Vaccine and Infectious Disease Division, Fred Hutchinson Cancer Research Center, Seattle, WA, 98109, USA

**Keywords:** Acellular pertussis vaccine, Diptheria-Tetanus-acellular Pertussis vaccine (DTaP), Disease surveillance, Ecological vaccine model, Endemic–epidemic model, Vaccination coverage

## Abstract

Routine vaccination with pertussis vaccines has been successful in driving down pertussis mortality and morbidity globally. Despite high vaccination coverage, countries such as Australia, USA, and UK have experienced increase in pertussis activity over the last few decades. This may be due to local pockets of low vaccination coverage that result in persistence of pertussis in the population and occasionally lead to large outbreaks. The objective of this study was to characterize the association between pertussis vaccination coverage and sociodemographic factors and pertussis incidence at the school district level in King County, Washington, USA. We used monthly pertussis incidence data for all ages reported to the Public Health Seattle and King County between January 1, 2010 and December 31, 2017 to obtain school district level pertussis incidence. We obtained immunization data from the Washington State Immunization Information System to estimate school-district level vaccination coverage as proportion of 19–35 month old children fully vaccinated with ≥4 doses of the Diphtheria-Tetanus-acellular-Pertussis (DTaP) vaccine in a school district. We used two methods to quantify the effects of vaccination coverage on pertussis incidence: an ecological vaccine model and an endemic–epidemic model. Even though the effect of vaccination is modeled differently in the two approaches, both models can be used to estimate the association between vaccination coverage and pertussis incidence. Using the ecological vaccine model, we estimated the vaccine effectiveness of 4 doses of Diphtheria-Tetanus-acellular-Pertussis vaccine to be 83% (95% credible interval: 63%, 95%). In the endemic–epidemic model, under-vaccination was statistically significantly associated with epidemic risk of pertussis (adjusted Relative Risk, aRR: 2.76; 95% confidence interval: 1.44, 16.6). Household size and median income were statistically significantly associated with endemic pertussis risk. The endemic–epidemic model suffers from ecological bias, whereas the ecological vaccine model provides less biased and more interpretable estimates of epidemiological parameters, such as DTaP vaccine effectiveness, for each school district.

## 1. Introduction

Pertussis is a highly transmissible infectious disease caused by bacterium *Bordetella pertussis* (Hamborsky et al., 2015; Rohani and Scarpino, 2019). There are currently two highly efficacious vaccines used worldwide — the Diphtheria-Tetanus-whole-cell-Pertussis (DTwP) and Diphtheria-Tetanus-acellular-Pertussis (DTaP) vaccines. A meta-analysis of randomized controlled trials and observational studies estimated the overall vaccine efficacy of DTaP vaccines to be 84% (95% confidence interval (CI): 81%, 87%) while that of DTwP to be 95% (95% CI: 88%, 97%) (Fulton et al., 2016). Global average vaccination coverage for the three-dose primary DTaP or DTwP vaccine series was 90% in 2019 in 125 countries as reported by the WHO (World Health Organization, 2020). However, despite high vaccination coverage with an effective vaccine, several countries have experienced a resurgence in pertussis (Pertussis Working group, 2014).

Aggregating estimates of vaccination coverage at the national or state level may hide local pockets of low vaccination. Sub-optimal local vaccination coverage could result in accumulation of susceptibles over time, and an introduction of a pertussis case in these areas could then result in an outbreak. Pockets of low vaccination coverage especially in areas of high population density could result in pertussis persistence in these areas (Broutin et al., 2004). Socio-demographic factors have also been important in shaping pertussis epidemiology. Even in high income countries, individuals who experience social and economic deprivation have lower access to community healthcare services resulting in delayed vaccination and poorer disease outcomes (Luman et al., 2005; Grant et al., 2003; Gilbert et al., 2017). It is of public health interest to investigate whether local disparities in vaccination coverage and socioeconomic factors are driving pertussis incidence.

Previous studies of effects of pertussis vaccination on pertussis incidence have shown heterogeneities in DTaP vaccination coverage at the local level and a significant association between area-level vaccination coverage and sociodemographic factors and area-level pertussis incidence (Duffy and Shea, 2012; Iroh Tam et al., 2015; Huang et al., 2017). However, these studies failed to account for the dependent nature of pertussis cases, and its complex, non-linear dynamics (Wakefield et al., 2019). Also, analysis of aggregated infectious disease data using ecological regression methods results in ecological bias where inference at the group level may not apply at the individual level (Richardson and Monfort, 2000; Wakefield, 2008). In this paper, we use methods that have been developed to analyze the association between area-level vaccination coverage and infectious disease incidence that address some of these issues using surveillance data marked in space and time (Fisher and Wakefield, 2020; Meyer et al., 2016).

Understanding the impact of community-level heterogeneity in vaccination coverage and social determinants of health on pertussis trends can help health authorities plan local interventions to prevent pertussis outbreaks. With this goal, we explored the association of area-level pertussis vaccination coverage with pertussis epidemicity and area-level socio-demographic factors with pertussis endemicity using surveillance, vaccination, and demographic data aggregated over school districts within King County, Washington, USA. We assessed this using an ecological vaccine model (Fisher and Wakefield, 2020) and an endemic-epidemic model (Meyer et al., 2016). Both approaches use multivariate time series of infectious disease data, can model occasional large outbreaks beyond regular endemic behavior, and can incorporate vaccination coverage as a covariate to study its effect on pertussis outbreaks.

## 2. Methods

### 2.1. Pertussis cases

Pertussis case data was obtained from the Public Health Seattle and King County (PHSKC) Department of Communicable Diseases and Immunizations. Pertussis is a nationally notifiable disease and cases are reported to PHSKC by primary care providers and diagnostic laboratories within 24 h of detection. The clinical case definition of pertussis used was a cough illness lasting 2 weeks or more with at least 1 of the following: paroxysms of coughing or inspiratory “whoop”, posttussive vomiting, or apnea (with or without cyanosis) for infants up to 1 year of age (CDC, 2014). Suspected, probable, and confirmed pertussis cases of all ages diagnosed in King County between January 1, 2010, and December 31, 2017 were included in this analysis. A confirmed case is defined as a case of acute cough illness of any duration with isolation of *B. pertussis* from a clinical specimen or polymerase chain reaction (PCR) positive for *B. pertussis*. A probable case is defined as (in absence of a more likely diagnosis) an illness meeting the clinical criteria, or an illness with cough of any duration with at least one of the clinical symptoms and contact with a laboratory confirmed case (epidemiologic link). Suspected pertussis cases are cases with cough lasting ≥ 2 weeks with no other symptoms, or cough of any duration with one of the casedefining symptoms without lab confirmation or epidemiologic link, or an epidemiologic link with cough of any duration and no other symptoms and no lab confirmation, or PCR positive for *B. pertussis* but no documentation of cough or case-defining symptoms (CDC, 2014; Washington State Department of Health, 2016). Demographic data (date of birth, gender, home address), date of diagnosis, vaccination status, number of DTaP doses received, and date of last DTaP dose was available for all reported cases.

### 2.2. Geocoding and aggregating pertussis cases at school-district level

**Fig. 1.**
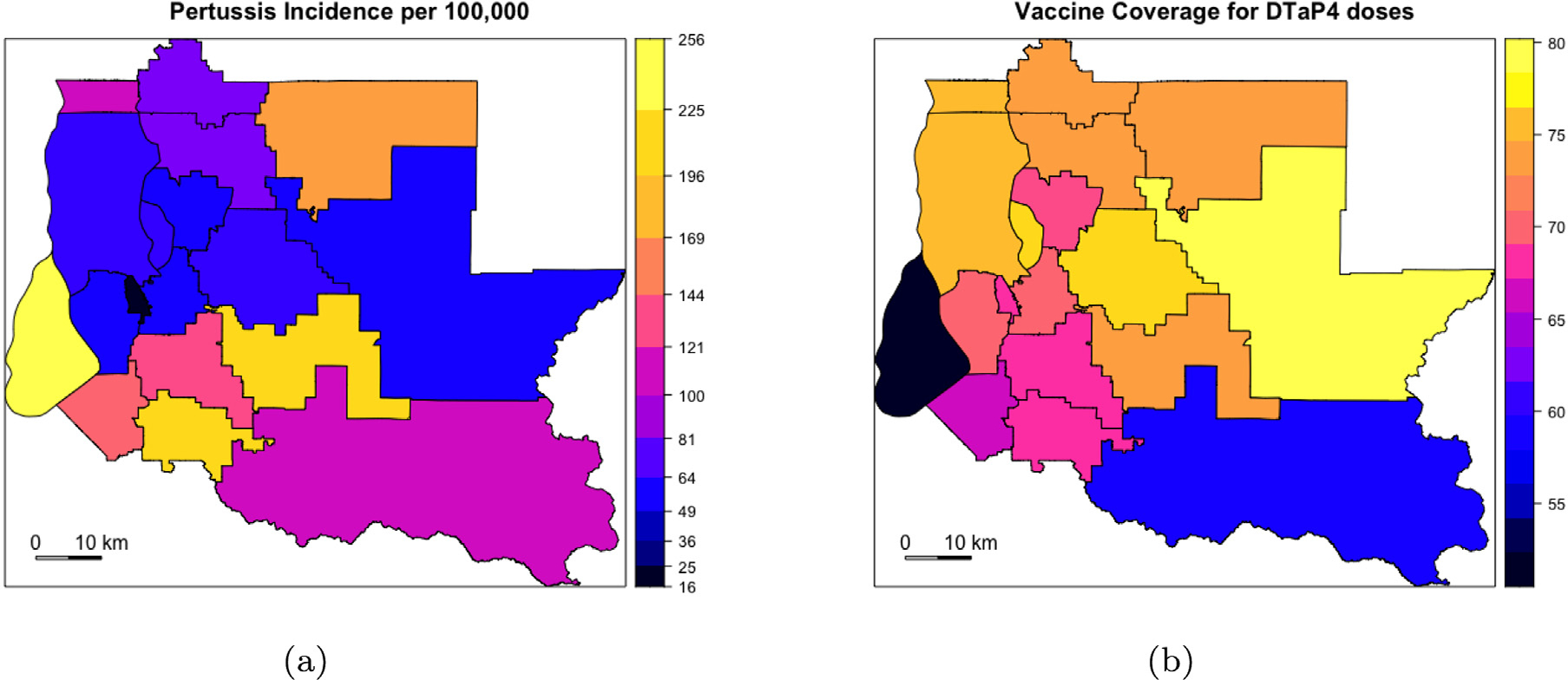
Pertussis incidence per 100,000 and DTaP vaccine coverage for ≥ 4 doses among children aged 19–35 months old in King County, Washington. (a) Total pertussis incidence between 2010 and 2017 plotted at school district level. Incidence is high in Vashon Island (in pale yellow in (a) and in black in (b) and school districts in southern King County; incidence is high in school districts in southern King County; (b) DTaP Vaccine coverage among children aged 19–35 months averaged over 8 years from 2011 to 2017 is plotted by school district. Visually, it appears that school districts with low vaccine coverage have high pertussis incidence.

We used ArcGIS 10.1 for geocoding cases’ home addresses (ESRI, 2012) and aggregated pertussis cases at the school district level. When the street address was missing or incorrect, we used zipcode for geocoding. School district level population estimates for the census year 2010 were obtained from the National Historical Geographical Information System (Appendix A Table 1) (Manson et al., 2017). A map of King County school districts (N=18) is in Appendix B Fig 1.

### 2.3. Estimating vaccination coverage at school district level

We obtained DTaP immunization records for children 0–9 years old born or living in King County, Washington between January 1, 2008, and December 31, 2017, from the Washington State Immunization Information System (WA-IIS). WA-IIS is a lifetime registry that tracks immunization records for people of all ages in Washington State (Washington State Department of Health, 2015). Healthcare providers such as primary care physicians, hospitals, and healthcare plans voluntarily report patient immunizations to the WA-IIS. Additionally, birth certificates of children born in King County are loaded into the registry fortnightly. The study cohort was restricted to children born in King County after 2008 to ensure data completeness and accuracy. Ninety-nine percent of children aged 4 months - 5 years have 2 or more immunizations recorded in the WA-IIS (Washington State Department of Health, 2015). Using WA-IIS data, we created a retrospective cohort of 316,404 children aged 3 months to 9 years. Vaccine name and date of receipt for all pediatric vaccines recommended from birth through 9 years of age was available for each child. Demographic information included date of birth, sex, current residential address, residential address at birth, county of residence, and insurance information. Home addresses (or zip codes when home addresses were incorrect or not available) of WA-IIS participants were geocoded by the WA Department of Health staff. We spatially overlaid the geocoded home addresses of WA-IIS participants onto a shapefile of King County school districts to obtain school district of residence for each child. DTaP doses are recommended at ages 2, 4, and 6 months of age, with booster doses at ages 15–18 months and 4–6 years (Havers et al., 2020). We used participants’ dates of birth and dates of DTaP vaccination to calculate age-appropriate vaccination status for each DTaP dose for each child. We estimated annual DTaP vaccination coverage at the school district level as proportion of 19–35 month old children living in a school district with ≥4 DTaP doses in a given year from 2011 to 2017 (the 2008 birth cohort turned 35 months old in 2011). We assumed that vaccine coverage in 2010 was the same as in 2011. We obtained school district level sociodemographic factors, namely, the proportion of population in each school district that are foreign born, White, non-citizens, speak a language other than English at home, proportion of population over 16 years old that have not completed high school, proportion of households with more than 4 people living in them, and median income, from the 2010 US Census data (U.S. Census Bureau, 2010). This study was reviewed and approved by the Washington State Institutional Review Board and PHSKC Research Administration Review Committee.

### 2.4. Notation

Here we present some notation and assumptions common to the ecological vaccine model and endemic–epidemic model. Let $Y_{it}$ and $N_{it}$ be the number of cases and the total population in school-district i at time t. Total population for King County summed over all school districts is given by N. Let $x_{it}$ be the time-varying vaccination coverage estimated as proportion of 19–35 month old children vaccinated against pertussis with ≥ 4 DTaP doses. Let $\lambda_{it}$ be the force of infection, i.e., risk of infection at time t for an individual who was susceptible at time t−1. For our analysis, we assume a time step of four weeks or a month which is the approximate generation time for pertussis (Vynnycky and White, 2010). Assuming a constant hazard rate between time steps, the probability that a susceptible individual at time t−1 gets infected at time t is given by $\lambda_{it}$. Assuming that time until infection is independent for all susceptible individuals (Halloran et al., 2010), the number of new cases in area i at time t can be modeled as:

(1) $$Y_{it}|\lambda_{it}∼Binomial\left(S_{i,t-1},1-e^{-\lambda_{it}}\right)$$

Assuming $\lambda_{it}$ is small, $1-e^{-\lambda_{it}}\approx \lambda_{it}$. When the number of infections is small and the population is large, a Poisson distribution approximates the Binomial distribution. Thus, eq. (1) can be written as $Y_{it}|\lambda_{it}∼Poisson\left(S_{i,t-1}\lambda_{it}\right)$. Let $\mu_{it}=S_{i,t-1}\lambda_{it}$, then a general form of eq. (1) is,

(2) $$Y_{it}|\mu_{it}∼Poisson\left(\mu_{it}\right)$$

Both the endemic–epidemic and ecological vaccine models assume that the number of infections is negligible compared to the number of susceptibles. Under this assumption, the number of susceptibles at time $t,\;S_{it}$, can be approximated by the initial number of susceptibles, thus $S_{it}∼N_{it}$. We assume that the population in school districts remains constant over time, i.e. $N_{it}∼N_{i}$. Because this is a partially vaccinated population, the initial number of susceptibles is given by $\left(1-x_{it}\right)N_{i}$ for the endemic–epidemic model and $\left(1-\phi x_{it}\right)N_{i}$ for the ecological vaccine model, where $x_{it}$ is the vaccine coverage in school district i at time t and ϕ is the direct vaccine effectiveness on susceptibility (reduction in individual’s risk of disease after vaccination). Thus, depletion of susceptibles is not explicitly modeled in either model. In the context of the ecological vaccine model, CI stands for credible intervals as this model uses the Bayesian framework. For the endemic–epidemic models, CI denotes confidence intervals.

### 2.5. The ecological vaccine model

Infectious disease surveillance data is often available aggregated over space and time. Using ecological regression models for infectious diseases data can be problematic because they do not account for the dependent nature of infectious disease data and can introduce ecological bias. The main feature of the ecological vaccine model developed by Fisher and Wakefield (Fisher and Wakefield, 2020) is that it reduces ecological bias when using area-level infectious disease data and its primary goal is inference. The authors started with an individual-level infectious disease model with vaccination as a parameter to model how vaccine reduces an individual’s risk of infection, under two modes of vaccine action: leaky and all-or-none (Halloran et al., 2010). In the leaky model, the individual’s risk of infection is reduced by a constant proportion for all vaccination individuals while in the all-or-none model (or primary vaccine failure), vaccinated individuals are fully protected against infection but the vaccine fails to take in some individuals. These individual-level vaccine models are then aggregated to area level to give ecological vaccine models. Under certain simplifying assumptions, the authors constructed an ecologically consistent model from an individual level model accounting for vaccine coverage. The detailed derivation of the ecological vaccine model can be found here (Fisher and Wakefield, 2020). The impact of vaccination in this model is defined as the ability of the vaccine to reduce susceptibility against infection. This ecological vaccine model is fit using the Bayesian framework in R package <monospace>rstan</monospace> (Guo et al., 2020) and provides posterior estimates and corresponding posterior credible intervals of epidemiologically relevant parameters.

In a partially vaccinated population, let ϕ be the reduction in a vaccine recipient’s risk of infection, which can be interpreted as the vaccine effectiveness after ≥ 4 DTaP vaccine doses (Fisher and Wakefield, 2020). Given that $x_{it}$ is the vaccine coverage in school district i at time t, the number of susceptibles in school district i can be written as $\left(1-\phi x_{it}\right)N_{i}$. Using the Bayesian framework, we can incorporate prior knowledge about pertussis vaccine effectiveness into the ecological vaccine model. Randomized controlled trials have estimated the vaccine efficacy of the DTaP vaccine to be ~ 85% (95% CI: 81%, 87% ) (Fulton et al., 2016). We fit the ecological vaccine model as:

(3) $$Y_{i,t+1}|\mu_{it},\phi ∼Poisson\left(N_{i}\left(1-\phi x_{it}\right)\left(\lambda_{i}\frac{Y_{it}}{N_{i}}+v_{it}\right)\right),$$

$$log\;\lambda_{i}=\alpha_{AR}+a_{i}$$

$$logv_{it}=\alpha_{EN}+b_{i}+\gamma \;sin\left(\omega_{t}\right)+\delta \;cos\left(\omega_{t}\right)-log(N)$$

$$a_{i}∼N\left(0,\sigma_{AR}^{2}\right)$$

$$b_{i}∼N\left(0,\sigma_{EN}^{2}\right)$$

ϕ∼Beta(c,d)

where $\mu_{it}$ is the total risk (epidemic plus endemic risk) and $v_{it}$ and $\lambda_{i}$ are the endemic and epidemic pertussis risk components. The school district specific random effects $a_{i}$ and $b_{i}$ are assumed to be independent; $\omega_{t}=\frac{2\pi t}{13}$; an informative beta prior Beta (10, 2.5) was used for ϕ with median 0.78 and 90% of the mass between 0.66 and 0.99. We assumed normal priors with mean 0 and variance 5 for $\alpha_{AR}$ and $\alpha_{EN}$ and variance 10 for γ and δ. We assumed frequency-dependent transmission in the formulation of $\lambda_{i}$ (Keeling and Rohani, 2008). Hamiltonian Monte Carlo sampling via R package <monospace>rstan</monospace> was used to fit this model (Guo et al., 2020). We adapted code published previously for an ecological vaccine model for measles data to include time-varying vaccination coverage (Fisher and Wakefield, 2020). As a sensitivity analysis, we fit the same model with non-informative priors on ϕ, with median 0.50 and 90% of the mass is between 0.05 and 0.95, to check the influence of priors on the estimate of vaccine effectiveness.

We also computed school-district specific time-varying autoregressive components and their 95% credible intervals from the ecological vaccine model. This parameter may be interpreted as the effective reproductive number, $R_{eff}$, which is defined as the average number of new cases per infectious case in a partially vaccinated population (Fisher and Wakefield, 2020). The time-varying autoregressive parameters were calculated as:

(4) $$(1-\overset{ˆ}{\phi }x_{it})\;exp\left({\overset{ˆ}{\alpha }}_{AR}+{\overset{ˆ}{a}}_{i}\right)$$

Fitted values were calculated as:

(5) $${\overset{ˆ}{Y}}_{it}=(1-\overset{ˆ}{\phi }x_{it})\left[exp\left({\hat{\alpha }}_{AR}+{\overset{ˆ}{a}}_{i}\right)Y_{i,t-1}+\left(\frac{N_{i}}{N}\right)exp\left({\overset{ˆ}{\alpha }}_{EN}+{\overset{ˆ}{b}}_{i}+\overset{ˆ}{\gamma }sin\left(\omega_{t}\right)+\overset{ˆ}{\delta }cos\left(\omega_{t}\right)\right)\right]$$

where $Y_{i,t-1}$ was the observed number of cases in school i and month t−1.

### 2.6. The endemic–epidemic model

The endemic–epidemic model is motivated by the Poisson branching process with immigration. Total pertussis incidence $\mu_{it}$ is split into two components: the endemic component with rate $v_{it}$ and the epidemic component with rate $\lambda_{it}Y_{it-1}$, such that $\mu_{it}=v_{it}+\lambda_{it}Y_{it-1}$, where $Y_{it-1}$ is the observed pertussis count in school district i and month t−1 (Held et al., 2005). The epidemic component can be further decomposed into the autoregressive and neighborhood components. In a model with spatial data, the autoregressive component models cases arising from infected individuals from the same area, while the neighborhood component captures cases arising from infected individuals in neighboring areas. The endemic component represents the background number of cases or remaining cases not explained by these two components. In this analysis, the endemic component captures incidence in an area related to sociodemographic factors. Each component can be modeled with a log linear model with covariates and fixed or random effects (Meyer et al., 2016). The model can be fit in the R <monospace>surveillance</monospace> package (Höhle et al., 2011) and likelihood estimation is done using the quasi-Newton algorithm. When random effects are included, penalized and marginal log-likelihoods are maximized alternately until convergence (Meyer et al., 2016).

In the simple model (Model 1), the endemic component included the population as an offset modeled as the fraction of the population that live in school district i denoted by $e_{i}$. To account for the temporal variance in incidence, the endemic component included an overall linear trend and a sinusoidal wave of frequency $\omega_{t}=\frac{2\pi t}{13}$. The endemic component is written as :

(6) $$\log\left(v_{it}\right)=\alpha_{EN}+\beta t+\gamma \sin\left(\omega_{t}\right)+\delta \cos\left(\omega_{t}\right),$$

where $v_{it}$ is the endemic risk of pertussis, $\alpha_{EN}$ is the endemic intercept, assumed to be constant over the region, and β is the parameter associated with temporal trend.

We included vaccination coverage in the epidemic component of the model as log proportion of children 19–35 months old that had fewer than 4 doses of DTaP vaccine because we are interested in the effect of pertussis vaccination on size and occurrence of pertussis epidemics. Effect of vaccination coverage on disease incidence has been modeled similarly for measles using the endemic–epidemic model before (Herzog et al., 2011). The epidemic component is written as:

(7) $$\log\left(\lambda_{it}\right)=\alpha_{AR}+\beta_{v}\log\left(1-x_{it}\right),$$

where $\lambda_{it}$ is the epidemic pertussis risk, $\alpha_{AR}$ is the epidemic intercept, $x_{it}$ is the vaccination coverage in school district i at time t, and $\beta_{v}$ is the parameter associated with under-vaccination. The proportion of 19–35 month old children under-vaccinated or susceptible in the school-districts is $1-x_{it}$. The intercept $\alpha_{AR}$ is assumed to be constant over all areas. In this simple endemic-epidemic model, we do not include a neighborhood component to be able to compare the $\alpha_{AR}$ and $\alpha_{EN}$ estimates with estimates from the ecological vaccine model. Overall, Model 1 can be written as:

(8) $$\mu_{it}=e_{i}v_{it}+\lambda_{it}Y_{i,t-1}$$

where $\mu_{it}$ is the total pertussis risk, $e_{i}$ is the population fraction in school district i used as a multiplicative offset in the endemic component, and $Y_{i,t-1}$ is the observed number of cases in school district i at time t−1.

We fit a separate endemic–epidemic model (Model 2) with sociodemographic covariates in the endemic component and the epidemic component split into autoregressive and neighborhood components to measure spatio-temporal dependence. School districts that shared a boundary were defined as neighbors. A matrix of transmission weights, $w_{ji}$, which represent the flow of cases from school district j to school district i when j≠i, was included in the neighborhood component. The model assumes that the epidemic can only arrive from adjacent areas. Thus, if two school districs share a boundary, the assigned weight was 1, otherwise 0. To reflect that people likely commute to densely populated metropolitan areas, we scaled the school district’s risk with respect to its population fraction, $e_{i}$. The neighborhood component can be written as:

(9) $$log\left(\zeta_{i}\right)=\alpha_{NE}+\beta_{\text{Pop}\;}log\left(e_{i}\right)$$

where $\zeta_{i}$ is the neighborhood associated risk of pertussis, $\alpha_{NE}$ is the intercept associated with the neighborhood component, and $\beta_{Pop}$ is the parameter associated with the population fraction of school-district $e_{i}$.

Let $\beta_{z}$ be a vector of parameters associated with a vector of school-district level sociodemographic covariates denoted by $\beta_{i}$, namely the proportion of population in each school district that are foreign born, White, non-citizens, speak a language other than English at home, proportion of population over 16 years old that have not completed high school, proportion of households with more than 4 people living in them, and median income. These factors included in the model were selected a priori based on previous research. Household size was selected as it could potentially impact pertussis transmission (Magpantay and Rohani, 2015). Median income, proportion of residents who were White, foreign-born, non-citizens, non-native English speakers, and who did not complete high school were selected as proxies for socioeconomic status and have been previously associated with vaccination coverage (Hill et al., 2016, 2018; Sánchez-González et al., 2017; Larson et al., 2014; Varan et al., 2017). Combining the endemic, epidemic, and neighborhood components, we get Model 2 as:

(10) $$\log\left(v_{it}\right)=\alpha_{EN}+\beta t+\gamma \sin\left(\omega_{t}\right)+\delta \cos\left(\omega_{t}\right)+\beta_{z}z_{i}\;\log\left(\lambda_{it}\right)=\alpha_{AR}+\beta_{v}\log\left(1-x_{it}\right)\;\;\log\left(\zeta_{i}\right)=\alpha_{NE}+\beta_{Pop}\log\left(e_{i}\right)\;\;\mu_{it}=e_{i}v_{it}+\lambda_{it}Y_{i,t-1}+\zeta_{i}\sum_{j\neq i}w_{ji}Y_{j,t-1}$$

where $Y_{j,t-1}$ is the number of cases in school-district j at time t−1.

## 3. Results

### 3.1. Descriptive analysis

Between 2010 and 2017, 1885 pertussis cases of all ages were reported in the 18 school districts in King County, WA. There was one large epidemic in 2012 with 894 cases and a smaller one in 2015 with 250 cases. The largest number of cases in a school district in a single month was 50 during the 2012 epidemic and occurred in Seattle school district, which also recorded the highest number of cases overall (*n* = 481). Because pertussis is a rare disease, many school districts recorded zero cases during several months. Tukwila school district recorded only 3 cases over the span of 10 years. Pertussis incidence per 100,000 by school district is in Fig. 1a.

Data from WA-IIS was used to estimate vaccine coverage at the school-district level which is displayed in Fig. 1b. Even though we used 19–35 month olds to estimate vaccination coverage with ≥ 4 DTaP doses, we assumed that the vaccine coverage for the entire population of King County is the same as the coverage estimated for this analysis. Vaccination coverage is higher in school districts in northern and northeastern King County, compared to school districts in the south. Vashon Island (in black) has the lowest vaccine coverage of all school districts. Within each school district, vaccine coverage appeared to increase with time between 2010 and 2017 (Table 1).

Pearson correlation coefficients showing correlations between DTaP vaccine coverage among 19–35 month old children and disease incidence calculated over a rolling 3-year window are in Table 2. With the exception of 2015, vaccine coverage with ≥ 4 doses of DTaP was negatively correlated with pertussis incidence. This association was strong and statistically significant in the years 2012 (*R*: −0.61, 95% CI: −0.20,−0.84), 2013 (*R*: −0.61, 95% CI: −0.19,−0.83) and 2014 (*R* ∶ −0.60, 95% CI: −0.19,−0.83). Thus, there is some indication that areas with higher vaccine coverage showed lower disease incidence, especially during a period of high incidence.

### 3.2. Ecological vaccine model

Summaries of posterior medians of fixed effects and 95% credible intervals from the ecological vaccine model are in Table 3. Using a strong prior for the vaccine effectiveness ϕ, we estimated it to be 83% (95% CI: 63%, 95%). Thus, the vaccine effectiveness associated with receiving ≥ 4 doses of DTaP compared to receiving < 4 DTaP doses is statistically significant. With a strong prior on ϕ, our results agree with estimates of efficacy of DTaP vaccine found in the literature (~85%) (Fulton et al., 2016). As a sensitivity analysis, we ran the same model using a uniform prior for ϕ and vaccine effect was estimated to be 79% (95% CI: 33%, 96%) (Appendix A Table 2). The uniform prior resulted in a slightly lower estimate for ϕ, but credible intervals were wider. This vaccine effectiveness estimate does not differentiate between primary vaccine failure and failure due to leakiness. With a uniform prior, the estimate of epidemic intercept $\alpha_{AR}$ in this model was also smaller with wider credible intervals. Using a uniform prior did not change the endemic intercept $\alpha_{EN}$ by much.

The school-district-specific time-varying $R_{\text{eff}}$ are plotted in Appendix C Fig 1. All estimates are below 1 and vary slightly with time within school-districts. There was no apparent effect of DTaP vaccine coverage on $R_{eff}$. Due to small number of cases within school-districts, the credible intervals of $R_{eff}$ were quite wide.

**Fig. 2.**
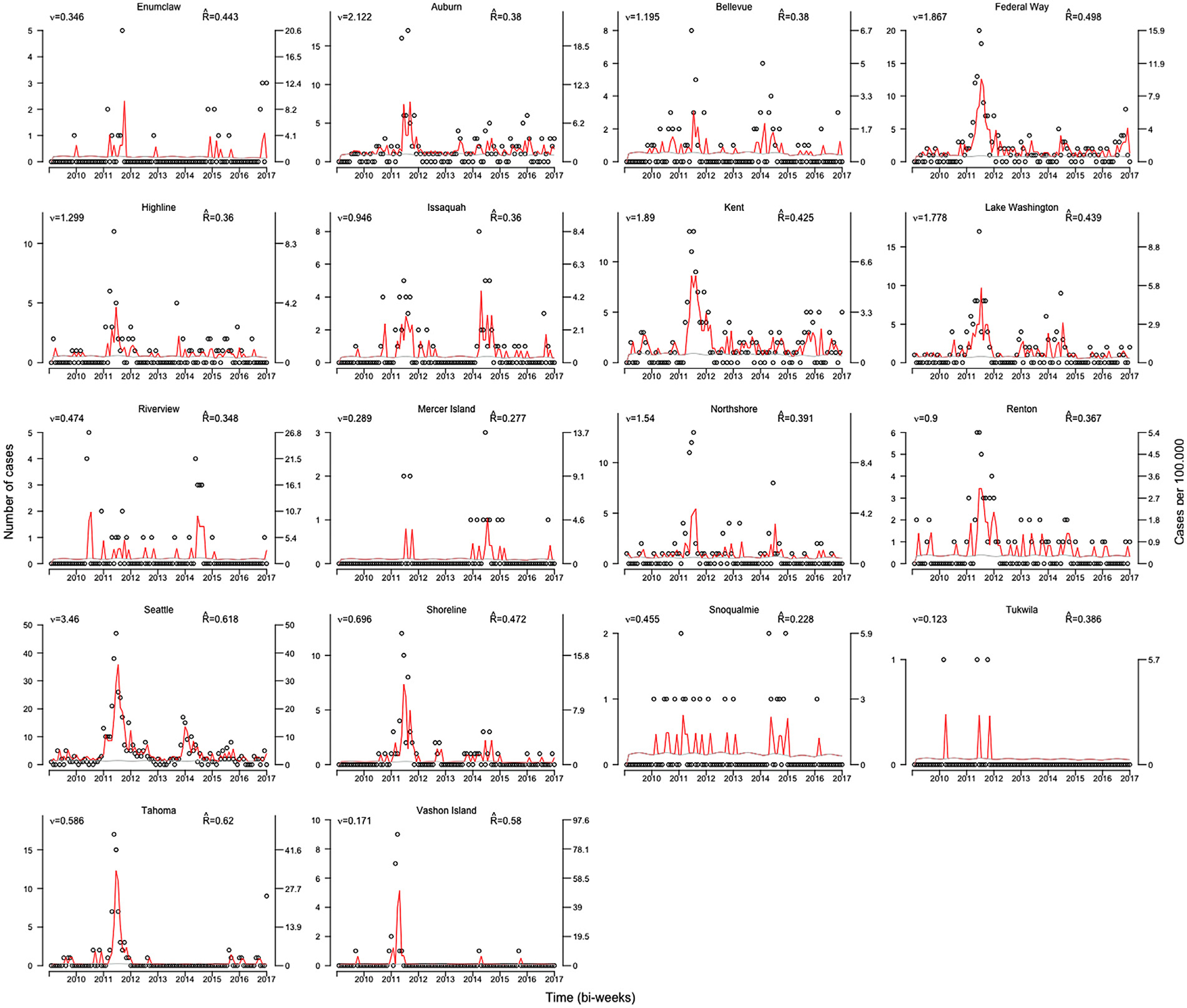
Ecological vaccine model fitted values by school district. Number of pertussis cases (left axis) and incidence per 100,000 (right axis) by school district. Left and right axes are on different scales for different school districts. Red lines are fitted epidemic component, gray lines show the endemic component, and black circles are absolute number of cases.

Fig. 2 shows the observed number of cases, incidence per 100,000 people, and model fits for each school district obtained from the ecological vaccine model. District-specific estimates of $R_{eff}$ and endemic risk $v_{it}$ are given in each panel. The model seems to fit the data well especially for areas with a large number of cases such as Federal Way, Kent, Lake Washington, and Seattle. The correlation coefficients between average DTaP vaccination coverage (measured as total children 19–35 months old with ≥ 4 doses of DTaP between 2010 and 2017 divided by total children 19–35 months old for each school district) and $R_{\text{eff}}$ (r=−0.05; 95% CI: −0.50, 0.42) and average DTaP vaccine coverage and endemic risk $v_{it}\;(r=0.18$; 95% CI: −0.31, 0.59) are small and not statistically significant. Population density (measured as persons per square mile) is positively correlated with both $R_{eff}\;(r=0.24$; 95% CI: −0.25, 0.63) and endemic risk (r=0.58; 95% CI: 0.16, 0.82). Thus, $R_{eff}$ in this study was not statistically significantly correlated with either vaccination coverage or population density. Endemic pertussis risk was significantly correlated with population density.

**Fig. 3.**
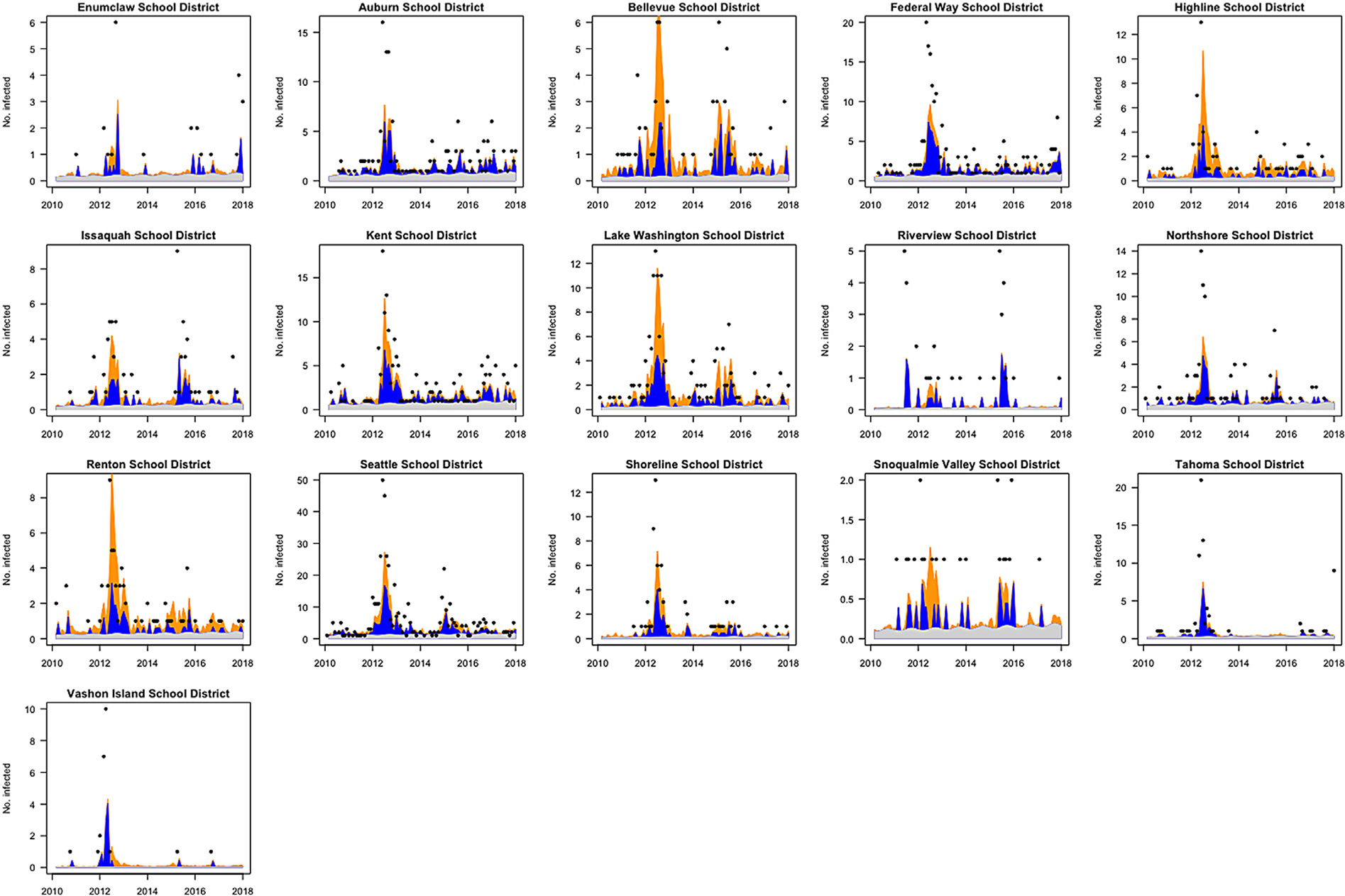
Endemic–epidemic model fitted values by school district. Endemic component is denoted in gray, autoregressive in dark blue and neighborhood component in yellow. Number of pertussis cases (left axis) and incidence per 100,000 (right axis) by school district. Left and right axes are on different scales for different school districts. Observed number of cases are displayed as black dots except for time points with zero cases. The model appears to fit the data well, especially for school districts with higher number of cases.

Appendix B Fig 2(a) and 2(b) show area-specific random effects of the autoregressive and endemic components. Areas with a large number of cases have larger autoregressive random effects. However, no such structure was found in the endemic random effects. There was no evidence of correlation between endemic and autoregressive random effects (Appendix B Fig. 3) which supports our decision to use independent random effects in our model.

### 3.3. Endemic–epidemic models

We will first discuss the results of the simple model with DTaP vaccination coverage in 19–35 month old children in the epidemic component as the only covariate and no neighborhood component or other demographic covariates included. The estimate of the exponentiated autoregressive intercept from this model ($\exp\left(\alpha_{AR}\right)=0.07$; 95% CI: 0.003, 1.43) is lower compared to the autoregressive intercept estimate from the ecological vaccine model ($exp\left(\alpha_{AR}\right)=1.1$; 95% CI: 0.75, 1.52) and has very wide confidence intervals (Table 4). The endemic estimate from the endemic-epidemic model (2.23; 95% CI: 1.99, 2.47) is lower than that from the ecological vaccine model (3.16; 95%CI: 2.77, 3.50). There is no comparable estimate to vaccine effect ϕ in this model. Here, the effect of DTaP vaccine coverage is estimated as $2^{exp\left(\beta_{v}\right)}$. It is interpreted as for each doubling of pertussis undervaccination rate (or doubling of susceptible population), the epidemic risk of pertussis increases multiplicatively by 3.54 fold (95% CI: 1.65, 23.05). Thus, this model suggests that epidemic pertussis risk was statistically significantly associated with DTaP vaccination coverage in 19–35 month old children. There is no strong temporal or seasonal trend in the data (Hill et al., 2016). A statistically significant overdispersion parameter suggests that using the negative binomial distribution was a more appropriate choice for this model than Poisson distribution.

We fit a second endemic–epidemic model where we split the epidemic component into the neighborhood and autoregressive components and added sociodemographic factors in the endemic component to estimate the effect of sociodemographic factors on endemic pertussis risk. For this model, for each doubling of under-vaccination rate (or doubling of susceptible population), the epidemic risk of pertussis increased multiplicatively by 2.76 fold higher (95% CI: 1.44, 16.6) (Table 4). This result is consistent with the under-vaccination coverage estimate from the simple model. Household size and median income were statistically significantly associated with endemic pertussis risk. For every unit increase in proportion of households with more than 4 individuals, the endemic risk of pertussis increased by 15% (95% CI: 8.3%, 20.9% ), adjusting for other covariates. For every $10,000 increase in median income, endemic pertussis risk decreased by 45% (95% CI: 25%, 60%). These are ecological associations and may not apply at the individual level. No significant association was found between pertussis risk and proportion of residents who were White, foreign born, non-citizens, spoke a language other than English at home, and had education less than high school.

The endemic–epidemic model appears to fit the data well (Fig. 3). According to this model, 32.8% of the time-averaged mean pertussis risk is explained by the endemic component, 34% by the autoregressive component, and 33% by the neighborhood component. The model suggests that a large proportion of cases in Renton, Lake Washington, and Bellevue school districts come from neighboring areas. This might be because these school districts have many neighbors and are also highly populated. On the other hand, Vashon Island, being an island (Appendix B Fig 1), has most of its incidence explained by the autoregressive component.

## 4. Discussion

In this study, we explored the relationship between local area-level vaccination coverage and sociodemographic factors with pertussis risk using statistical models that account for the non-linear dynamics and dependent nature of infectious diseases and address ecological bias. From the endemic–epidemic model, we found that under-vaccination at the school-district level was significantly associated with pertussis epidemics and household-size and median income were associated with endemic pertussis risk. Results from the ecological vaccine model also showed that vaccination is highly effective in preventing pertussis, while providing epidemiologically interesting parameters such as vaccine effectiveness and effective reproduction number. Our findings emphasize the need to monitor sub-county level DTaP vaccination coverage to assist local health authorities such as school boards, community health clinics, and public health officials target interventions to most affected areas.

We estimated the vaccine effectiveness after 4 doses of DTaP vaccine to be 83%, which is commensurate with what is known in the literature (Fulton et al., 2016). We also found local (school-district level) variations in vaccination coverage that were associated with pertussis epidemics, similar to other studies in the U.S (Atwell et al., 2013; Omer et al., 2008). This suggests that health jurisdictions should attempt to monitor vaccination uptake at sub-county levels in addition to state and county levels to design optimal vaccination strategies. Of the sociodemographic factors assessed, we found that median income and household size were associated with endemic pertussis risk. Low income has been associated with lower vaccine coverage and increased likelihood of disease risk in the US (Hill et al., 2016; Larson et al., 2014). Household size and crowding have been identified as important factors contributing to pertussis transmission (Magpantay and Rohani, 2015; Saeidpour et al., 2022). In large households, there is an increased possibility of prolonged close contact and transmission between an index cases and household members. Other studies have found higher pertussis incidence and lower vaccination coverage among populations with longstanding barriers to healthcare access and greater social inequities, such as racial and ethnic minorities, migrant populations, and populations with lower education attainment (Sánchez-González et al., 2017; Wolf et al., 2016; Gilbert et al., 2017; Varan et al., 2017; Hill et al., 2016). Even though we did not observe statistically significant associations between these sociodemographic factors and pertussis risk in our study, it is critical to provide accessible vaccination and primary healthcare without financial barriers to these populations.

The strengths of the endemic–epidemic models over a Poisson regression model for infectious disease data are that the endemic–epidemic models can deal with multivariate time-series data and cope with the occasional large outbreak, in addition to incorporating covariates (Herzog et al., 2011). Moreover, they are easily fit and allow modeling of neighborhood effects to study spatio-temporal dependence. endemic–epidemic models with various complexities have been used to study measles (Herzog et al., 2011), meningococcal disease (Meyer et al., 2012; Meyer and Held, 2014), psychiatric hospital admissions (Meyer et al., 2012), and Norovirus infections (Meyer and Held, 2017).

An important limitation of the endemic–epidemic model is that they may not be suitable for individual-level inference. Endemic–epidemic models were developed with disease prediction as their main goal and do not address ecological bias. It has been shown before that for estimating effects of vaccination on infectious disease incidence, endemic–epidemic models can give biased estimates for the epidemic and endemic risks (Fisher and Wakefield, 2020). Thus, effects of covariates obtained from these models should be interpreted carefully, explicitly stating ecological bias as a drawback. In the endemic–epidemic models, vaccination coverage (and other covariates) can be included in either the epidemic or endemic components. In earlier studies, this decision was driven by which model formulation fit the data best and not with inference as the primary goal. In eq. (7), given how vaccination coverage is included in the form of $log\left(1-x_{it}\right)$, the parameter associated with vaccination coverage is the flexibility parameter that improves model fit (Fisher and Wakefield, 2020). The interpretation of this parameter as expected multiplicative change in disease risk for every 2 fold increase in undervaccination or susceptibility is difficult to interpret and non-intuitive. In our study, the decision to include vaccination coverage in the epidemic component was driven by the hypothesis that lower DTaP vaccination coverage could result in pertussis outbreaks.

Like the endemic–epidemic model, the ecological vaccine model also accounts for dependency of outcomes for infectious disease models and allows for occasional large outbreaks. Compared to the endemic–epidemic model, the ecological vaccine model more appropriately models aggregated infectious disease data. It gives less biased and easily interpretable estimates of epidemic and endemic risks under certain assumptions. One might consider using ecological vaccine models over endemic–epidemic models when individual-level inference is the goal of the analysis.

The ecological vaccine model also has certain limitations. The model needs to be developed further to include neighborhood structure and variable infectious periods (Fisher and Wakefield, 2020). We assumed that the DTaP vaccination coverage for the entire population of King County is the same as that estimated using immunization data for 19–35 month old children. A similar assumption was made in other studies on effect of measles vaccination coverage on measles incidence in Germany (Fisher and Wakefield, 2020; Herzog et al., 2011). We also assumed (in both models) that the population of King County remains constant, which is a strong assumption. Neither model explicitly accounts for depletion of susceptible population or gives insights into the mechanism of vaccine failure.

In summary, we found that school-district level low DTaP vaccination coverage among 19–35 month old children was statistically significantly associated with pertussis epidemicity, and median income and household size were associated with endemic pertussis risk in King County, WA. Even though we estimated high DTaP vaccine effectiveness after four doses, local pockets of low vaccination coverage could result in occasional pertussis outbreaks. Researchers should consider using the ecological vaccine model when individual-level vaccination and disease status is not available, but area-level vaccine coverage and disease incidence are known to get less biased estimates of vaccine effectiveness.

## Supplementary Material

1

## Figures and Tables

**Table 1:** Pertussis cases and estimated DTaP vaccination coverage among children aged 19–35 months^a^ in King County, Washington, between 2010 and 2017.

School district	Pop^e^	Pertussis cases		Estimated vaccine coverage^d^ (%)						
	
		Max cases^b^	Sum cases^c^	2011	2012	2013	2014	2015	2016	2017

Enumclaw	25 179	6	26	51.7	54.3	58.9	55.6	58.1	69.2	70.6
Auburn	84 053	16	165	67.6	68.4	69.1	66.3	66.8	70.8	68.7
Bellevue	124 003	6	71	66.4	68.2	67.9	65.3	69.1	80.1	75.8
Federal Way	130 706	20	215	64	67.2	67.6	67.0	65.0	73.4	70.1
Highline	124 481	13	78	70.9	69.0	73.2	72.7	73.4	80.5	76.7
Issaquah	98 660	9	73	76.4	77.1	76.6	74.2	76.6	82.6	79.8
Kent	158 233	18	195	65.8	67.4	67.5	69.2	70.7	77.8	73.1
Lake	177 476	13	152	69.5	71.1	70.9	71.0	74.1	82.5	77.3
Washington										
Riverview	19 315	5	36	74.9	73.9	70.0	71.5	69.1	79.4	75.3
Mercer Island	22 699	2	15	76.8	76.7	78.9	73.8	80.4	84.9	79.3
Northshore	122 684	14	101	72.8	74.6	75.6	72.3	70.2	80.1	74.0
Renton	115 511	9	73	70.9	71.7	73.2	72.1	72.0	77.4	74.6
Seattle	609 471	50	481	71.1	73.5	74.4	73.7	74.8	83.8	81.8
Shoreline	65 547	13	72	73.2	75.9	75.8	74.6	72.0	86.1	85.1
Snoqualmie	35 054	2	22	76.6	77.3	75.1	76.2	80.1	86.0	82.1
Valley										
Tukwila	18 038	1	3	64.3	66.1	67.7	68.4	69.5	75.2	70.1
Tahoma	37 376	21	83	70.1	75.2	73.0	70.2	69.8	81.0	78.7
Vashon	10 624	10	24	51.0	48.3	62.6	45.1	49.1	65.3	55.1
Island										

a Vaccination coverage defined as proportion of 19–35 month old children with ≥4 DTaP doses.

b Max Cases are the maximum number of cases in any given month.

c Sum Cases are the total number of cases between 2010 and 2017.

d Vaccination coverage of 2010 was assumed to be the same as 2011.

e Total Population in census year 2010.

**Table 2:** Estimated Pearson’s correlation coefficients and 95% confidence intervals (CI) between pertussis incidence and estimated DTaP vaccination coverage among children aged 19–35 months in a rolling 3-year window.

Year	Pearson’s correlation coefficient	95% CI	P-value

2012	−0.61	(−0.20, −0.84)	0.006
2013	−0.61	(−0.19, −0.83)	0.007
2014	−0.60	(−0.19, −0.83)	0.008
2015	0.19	(−0.29, 0.61)	0.4
2016	−0.02	(−0.48, 0.45)	0.9
2017	−0.20	(−0.61, 0.28)	0.4

**Table 3:** Posterior median estimates and 95% credible intervals (CI) from ecological vaccine model with a strong prior on vaccine effectiveness ϕ, ϕ∼Beta(10,2.5).

Parameter	Parameter description	Posterior medians	95% CI

$\alpha_{AR}$	Epidemic intercept	0.10	(−0.28, 0.42)
ϕ	Vaccine effect	0.83	( 0.63, 0.95)
$\alpha_{EN}$	Endemic intercept	3.16	( 2.77, 3.50)
γ	Seasonality term	−0.02	(−0.13, 0.09)
δ	Seasonality term	−0.09	(−0.21, 0.02)
$\sigma_{AR}$	Variance of $\alpha_{AR}$	0.38	( 0.22, 0.66)
$\sigma_{EN}$	Variance of $\alpha_{EN}$	0.45	( 0.30, 0.71)
$exp\left(\alpha_{AR}\right)$		1.10	( 0.75, 1.52)

**Table 4:** Results from endemic-epidemic models for effect of DTaP vaccination coverage in 19–35 month old children and other sociodemographic factors on pertussis risk.

Parameters	Epidemic component		Endemic component		log (L)	p	AIC
	β Coefficient	95% CI	β Coefficient	95% CI			

Model 1					−1939.89	7	3893.78

$a_{AR}$	−2.70	−5.78, 0.36					
log(Under-vaccination)	0.59	−0.32, 1.51					
$a_{EN}$			2.23	1.99, 2.47			
Temporal trend			0.002	−0.001, 0.006			
γ			−0.04	−0.20, 0.11			
δ			−0.02	−0.18,0.13			

Model 2					-1859.43	16	3750.85

$a_{AR}$	−2.42	−5.8, 0.98					
log(Under-vaccination)	0.38	−0.63, 1.4					
$a_{NE}$	−0.74	−1.62, 0.14					
log(population fraction)	0.72	0.47, 0.97					
$a_{EN}$			7.69	−2.29, 17.66			
Temporal trend			0.005	0.0009, 0.01			
γ			−0.09	−0.31, 0.12			
δ			−0.09	−0.31, 0.11			
% Foreign born			−0.09	−0.34, 0.13			
% White race			−0.03	−0.13, 0.007			
% Not Citizens			0.08	−0.15, 0.31			
% with Household size ≥ 4			0.14	0.08, 0.19			
% language other than English at home			−0.03	−0.38, 0.31			
% education less than high school			−0.2	−0.40, 0.009			
Median income (for every $10,000)			−0.61	−0.93, −0.29			

log(L): Log likelihood; p: number of parameters ; Akaike’s Information Criterion is calculated as (*AIC*) = 2*p* – 2*log*(*L*); lower AIC values are preferred.

Model 1: DTaP vaccination coverage in epidemic component, no neighborhood component.

Model 2: DTaP vaccination coverage in epidemic component, simple neighborhood structure, sociodemographic covariates.
