# Supplementary material for: Association between pertussis vaccination coverage and other sociodemographic factors and pertussis incidence using surveillance data
Source: Epidemics. Author manuscript; Available in PMC 2023 Oct 18. (PMC10584035; doi:10.1016/j.epidem.2023.100689)
Supplement: 1 [file NIHMS1929374-supplement-1.pdf]

## Appendix A. Appendix Tables

Table 1: Demographic characteristics of King County school districts, 2010-2017

| **School district** | **% Caucasian** | **% Foreign Born** | **% Non-citizens** | **% Households with $\geq$ 4 people** | **%Median Income (USD)** | **% with less than High School education** | **% Non-English speakers** |
|---|---|---|---|---|---|---|---|
| Enumclaw | 91.7 | 3.3 | 2.0 | 23.2 | 68900 | 5.8 | 1.56 |
| Auburn | 70.9 | 17.7 | 10.9 | 27.2 | 55442 | 11.0 | 11.1 |
| Bellevue | 63.6 | 30.4 | 18.1 | 20.9 | 81656 | 3.7 | 14.4 |
| Federal Way | 60.4 | 21.3 | 11.5 | 27.3 | 61157 | 10.6 | 13.7 |
| Highline | 56.7 | 23.7 | 13.3 | 24.4 | 50946 | 16.2 | 17.3 |
| Issaquah | 73.9 | 19.6 | 10.6 | 28.3 | 101139 | 2.1 | 8.1 |
| Kent | 61.8 | 20.8 | 11.3 | 28.7 | 65808 | 10.7 | 14.4 |
| Lake Washington | 74.5 | 21.5 | 13.9 | 22.4 | 93294 | 3.2 | 7.7 |
| Riverview | 90.1 | 7.7 | 4.5 | 29.3 | 98248 | 5.1 | 4.1 |
| Mercer Island | 77.8 | 16.9 | 6.5 | 23.4 | 120994 | 0.7 | 4.2 |
| Northshore | 80.0 | 13.6 | 7.1 | 25.7 | 87473 | 3.5 | 6.0 |
| Renton | 53.2 | 24.9 | 12.3 | 23.1 | 62655 | 10.3 | 16.7 |
| Seattle | 69.4 | 16.9 | 8.1 | 13.2 | 60645 | 6.0 | 9.9 |
| Shoreline | 73.6 | 18.0 | 7.0 | 20.3 | 71020 | 5.8 | 9.2 |
| Snoqualmie Valley | 88.9 | 7.4 | 4.3 | 30.0 | 100038 | 3.9 | 3.2 |
| Tukwila | 43.4 | 35.5 | 23.7 | 25.8 | 41675 | 23.0 | 26.7 |
| Tahoma | 87.7 | 5.9 | 3.0 | 31.5 | 92596 | 4.3 | 3.2 |
| Vashon Island | 92.3 | 3.4 | 1.7 | 17.0 | 80000 | 2.6 | 0.7 |

Table 2: Posterior median estimates and 95% credible intervals (CI) from ecological vaccine model with a uniform prior on vaccine effect $\phi$, $\phi \sim Beta(1,1)$

| **Parameter** | **Parameter Description** | **Posterior medians** | **95% CI** |
|---|---|---|---|
| $\alpha_{AR}$ | Epidemic intercept | 0.03 | $-0.56$, 0.43 |
| $\phi$ | Vaccine effect | 0.79 | 0.33, 0.96 |
| $\alpha_{EN}$ | Endemic intercept | 3.09 | 2.47, 3.51 |
| $\gamma$ | Seasonality term | $-0.02$ | $-0.13$, 0.09 |
| $\delta$ | Seasonality term | $-0.09$ | $-0.21$, 0.02 |
| $\sigma_{AR}$ | Variance of $\alpha_{AR}$ | 0.37 | 0.21, 0.66 |
| $\sigma_{EN}$ | Variance of $\alpha_{EN}$ | 0.46 | 0.30, 0.73 |
| $\exp(\alpha_{AR})$ | | 1.03 | 0.57, 1.54 |

## Appendix B. Appendix Figures

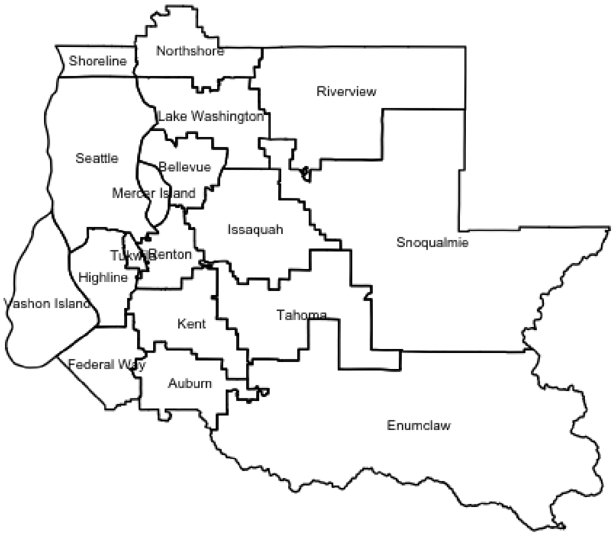


Fig 1: Map of school districts in King County, Washington

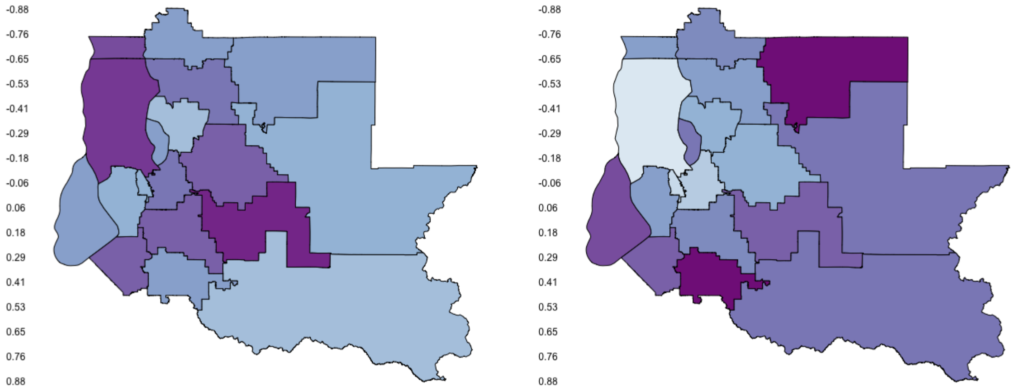


(a) (b)

Fig 2: Maps of random effects of autoregressive (a) and endemic components (b) from ecological vaccine model

There appears to be some spatial structure in autoregressive random effects but not in endemic random effects.

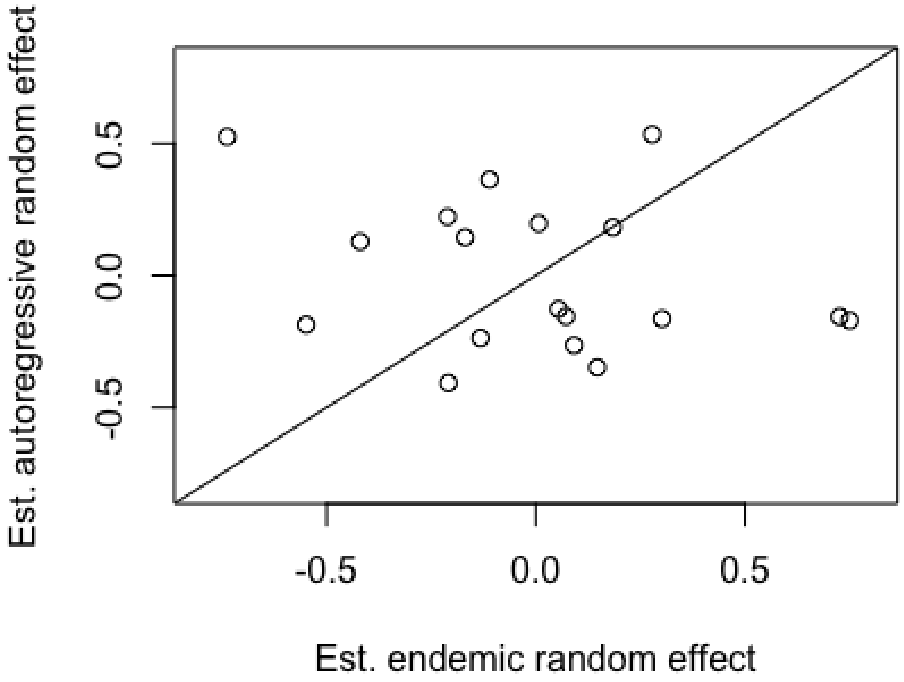


Fig 3: Correlation between random effects from ecological vaccine model

## Appendix C. Effective Reproduction number of pertussis using the ecological vaccine model

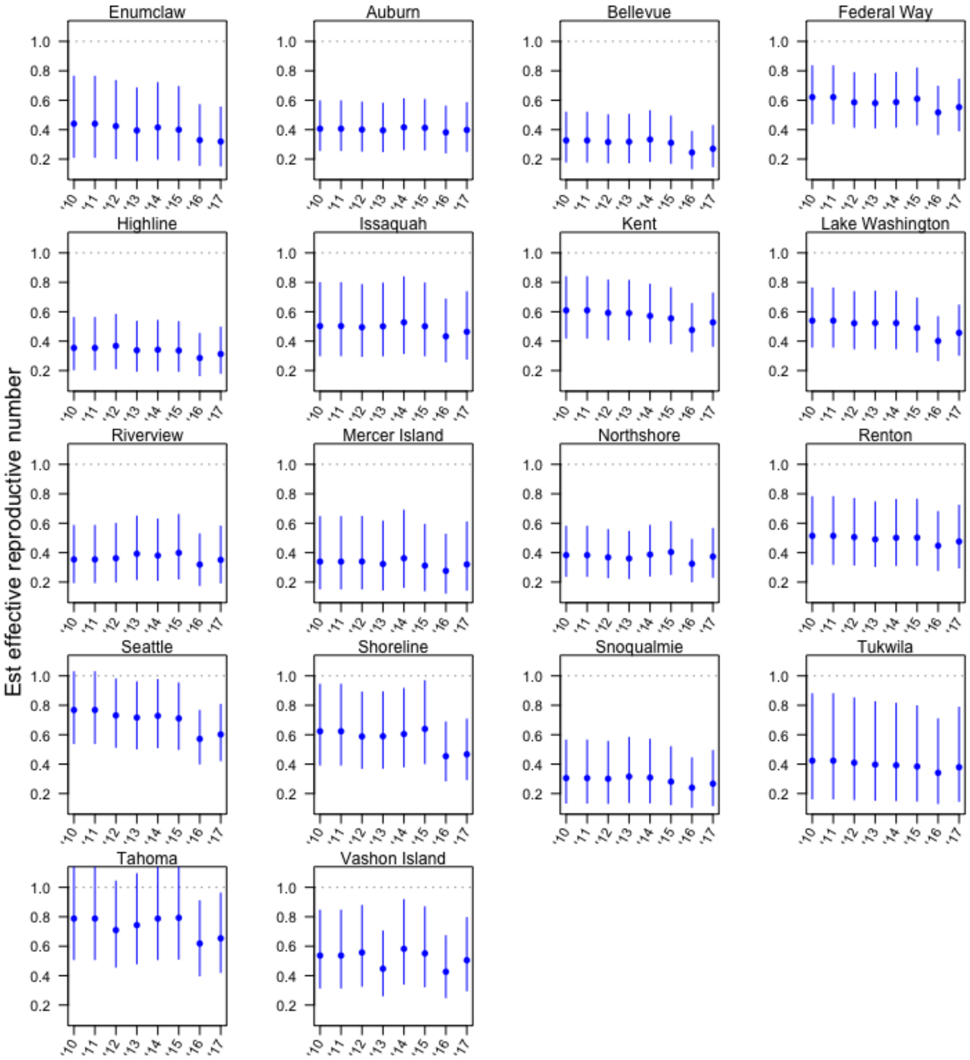


Figure 1: Estimated school-district-level autoregressive components ($R_{eff}$) and associated 95% Credible Intervals estimated using ecological vaccine model

Effective reproductive number ($R_{eff}$) is the average number of secondary

cases caused by an infectious case in a partially immune (due to prior infection or vaccination) population. $R_{eff}$, computed as $(1 - \phi)x_{it}exp(\alpha_{AR} + \alpha_i)$ varies with time within each school district, reflecting changes in vaccine coverage over time within school districts. Credible intervals for $R_{eff}$ are larger for areas with fewer cases. Some school districts with high vaccine coverage have low estimates of $R_{eff}$ (Bellevue, Mercer Island), but there is no clear effect of vaccine coverage on the autoregressive component. On the other hand, school districts with large population sizes had higher $R_{eff}$ estimates(Seattle, Kent, Lake Washington, Federal Way)

Similar to the $R_{eff}$ estimates for measles in the Fisher et al. study [1], the estimates of $R_{eff}$ from our model were lower than 1. This may be because King County only experienced one large and one small outbreak of pertussis in the 8 year study period, and the estimate of the autoregressive component $\hat{\alpha}_{AR}$ was averaged over the entire period. Another explanation for low $R_{eff}$ estimates in this study might be that pertussis is severely underreported, with $\sim 10\%$ of cases reported [2] [3]. Discretization of time into larger bins (months) can also result in biased estimates of reproductive numbers [4]. Thus, the ecological vaccine model may be limited in its ability to estimate $R_{eff}$.

## References

[1] Leigh H. Fisher and Jon Wakefield. Ecological inference for infectious disease data, with application to vaccination strategies. *Statistics in Medicine*, 39(3):220–238, 2020.

[2] Rubén Solano, Inma Crespo, María Isabel Fernández, Carles Valero,

María Isabel Álvarez, Pere Godoy, Joan A. Caylà, and Àngela Domínguez. Underdetection and underreporting of pertussis in children attended in primary health care centers: Do surveillance systems require improvement? *American Journal of Infection Control*, 44(11):e251–e256, 2016.

[3] Pejman Rohani and Samuel V. Scarpino. *Pertussis Epidemiology, Immunology, and Evolution*. Oxford University Press, 1 edition, 2019.

[4] Matthew J Ferrari, Ottar Bjornstad, and Andrew Dobson. Estimation and inference of R 0 of an infectious pathogen by a removal method. 198:14–26, 2005.
